# Assessing posttraumatic stress disorder in children with mild to borderline intellectual disabilities

**DOI:** 10.3402/ejpt.v7.29786

**Published:** 2016-01-11

**Authors:** Liesbeth Mevissen, Robert Didden, Hubert Korzilius, Ad de Jongh

**Affiliations:** 1 Department De Swaai Youth, Mental Health Organisation (MHO) GGZ Friesland, Drachten, The Netherlands; 2 Behavioural Science Institute, Radboud University Nijmegen, Nijmegen, The Netherlands; 3 Institute for Management Research, Radboud University, Nijmegen, The Netherlands; 4 Department of Behavioral Sciences, Academic Centre for Dentistry Amsterdam (ACTA), University of Amsterdam and VU University Amsterdam, Amsterdam, The Netherlands; 5 School of Health Sciences, Salford University, Manchester, United Kingdom

**Keywords:** Trauma, PTSD, intellectual disabilities, diagnosis, assessment, children, ADIS-C, caregivers, A1 criterion, DSM-IV-TR, DSM-5

## Abstract

**Background:**

Evidence suggests that children with mild to borderline intellectual disabilities (MBID; IQ 50–85) have an elevated risk for both being exposed to potentially traumatic events and developing a posttraumatic stress disorder (PTSD). In this target group, PTSD often remains undiscovered due to a lack of diagnostic instruments. Valid instruments for the assessment of PTSD in children with MBID are therefore needed.

**Objective:**

The aim of the current study was to validate the adapted PTSD section of the Anxiety Disorders Interview Schedule for Children (ADIS-C) for the assessment of PTSD in children with MBID according to DSM-IV-TR and DSM-5 criteria.

**Method:**

Eighty children (aged 6–18 years) with MBID who were referred to an outpatient psychiatric service and their primary caregivers were interviewed using the adapted ADIS-C.

**Results:**

The adapted ADIS-C PTSD section has excellent interrater reliability and good convergent validity. PTSD symptoms described spontaneously by children with MBID and their caregivers closely matched those included in the DSM-IV-TR and DSM-5. Many of the children who met Criterion A did not meet PTSD symptom criteria. Conversely, children meeting the full PTSD criteria were more likely than other children with MBID to have been exposed to at least one traumatic event meeting Criterion A and to a higher total number of potentially traumatic events.

**Conclusions:**

The results support the reliability and validity of the adapted ADIS-C PTSD section for assessing PTSD in children with MBID. The use of this clinical interview helps to improve detection of PTSD and subsequent access to trauma-focused interventions for this at risk target group.

Exposure to severe adverse events such as interpersonal violence, sexual abuse, a severe accident, or the sudden loss of a loved one can have a far-reaching impact on someone's life. Up to 80% of people encounter such potentially traumatic events and a significant proportion of about 7% develop posttraumatic stress disorder (PTSD) (Kessler et al., 2005). PTSD is associated with clinically significant distress or impairment in social, occupational, and other important areas of daily functioning in both adults and children (Yule, 2001). The events may also lead to other symptoms and conditions, for example, major depression, anxiety disorders, substance use disorder, and physical health problems (Olff, 2015). Especially, the experience of severe, prolonged, or repeated stressors, such as child abuse or interpersonal violence, is associated with chronic mental and physical health problems involving high costs to society, thereby assigning great importance to timely trauma detection and subsequent trauma treatment (Olff, 2015).

## Intellectual disability a risk factor for PTSD

Children with intellectual disabilities (ID) have been found to experience a greater number and range of adverse life events than children without ID (Hatton & Emerson, 2004). Furthermore, they have an elevated risk of developing psychiatric, emotional, or conduct disorders (Hatton & Emerson, 2004). Also, evidence suggests that children with ID suffer from more severe forms of psychopathology than children without ID (Hatton & Emerson, 2004). Although cognitive and adaptive impairments are supposed to be a risk factor for the development of PTSD (DiGangi et al., 2013), very few studies have been conducted on the manifestations of PTSD, and the development and psychometric evaluation of instruments for the assessment of PTSD in children with ID (Mevissen & De Jongh, 2010).

## Assessment of PTSD in ID

To facilitate assessment of PTSD in children with mild to borderline ID (MBID), the present study replicates and extends the study by Mevissen, Barnhoorn, Didden, Korzilius, and De Jongh (2014) that examined the feasibility of an adapted version of the PTSD clinical interview (Anxiety Disorders Interview Schedule for Children [ADIS-C] PTSD section) in a sample of children with MBID (IQ 50–85). The latter study explored to what extent manifestations of PTSD corresponded with four PTSD algorithms, that is, DSM-IV-TR, DSM-5 proposed revision, PTSD-Alternative Algorithm, and the proposed DSM-5 for preschool children. The adapted ADIS-C PTSD appeared applicable for children with MBID and the study's findings suggested that manifestations of PTSD correspond with the four PTSD algorithms that were developed based on research in people without ID (Mevissen et al., 2014).

## The current study

The aim of the current study was to validate the adapted ADIS-C PTSD using a sample of children with MBID including the children's caregivers. To this end, its reliability as well as its convergent validity was determined. It was hypothesized that (1) PTSD symptoms in children with MBID would correspond with those included in the DSM-IV-TR (American Psychiatric Association, 1994) and DSM-5 (American Psychiatric Association, 2013) PTSD algorithms, (2) fulfilling Criterion A for trauma would be associated with the presence of PTSD, (3) children meeting PTSD symptom criteria would report higher subjective levels of daily life impairments than children not meeting criteria for PTSD, (4) children meeting PTSD symptom criteria would report a higher level of exposure to potentially traumatic events than those not meeting PTSD symptom criteria and, finally, given that children exposed to trauma have high rates of psychiatric disorders (Copeland, Keeler, Angold, & Costello, 2007), (5) positive correlations would be found between rates of PTSD symptoms and Child Behavior Checklist (CBCL) internalizing, externalizing, and total scale scores.

## Method

### Participants

Participants were 80 children (46% [*n=*37] female) with MBID who were referred to an outpatient center for child and adolescent psychiatry in the eastern part of the Netherlands. Their mean age was 11.6 years (min=6, max=18, SD=3.25). Of this sample, 41% (*n=*33) had mild ID (IQ 50–70), and 59% (*n=*47) had borderline ID (IQ 70–85). Mean IQ was 72 (min=51, max=84, SD=8.00).

Their primary caregivers also participated. For 53 children, the primary caregiver was the mother; for 5 children, it was the father; and for 17 children, it was both parents, whereas in the remaining cases, adoptive parents (*n=*1), a legal guardian (*n=*2), or a professional caregiver (*n=*2) participated. Ninety percent (*n=*72) of the children lived at home, 10% (*n=*8) lived in a residential facility.

### Measures

#### Adapted ADIS-C PTSD section

The adapted ADIS-C PTSD section (Mevissen et al., 2014) uses simplified language and visual cues. The interview consists of an event and a symptom section with answer categories “yes,” “no,” or “other.” The event section (26 items) includes type A trauma events as well as life events and has one open-ended question. Events the child had been exposed to are visualized on a timeline to help the child keep in mind the events when symptoms are asked for. The symptom section (37 items) includes 30 symptoms originating from PTSD measures that are used in children without ID, and five potentially atypical symptoms that were found in the literature on clinical experiences regarding PTSD and its treatment in people with ID. Also, two open-ended questions are part of the symptom section (question 37 child/caregiver): “If something reminds you/your child of the event(s), have you noticed anything that's different about yourself/your child?” and the last symptom question: “Have you noticed anything else that's different about yourself/your child since the event(s)?” If the answer is “yes,” the interviewer asks “What do you notice?” Finally, a thermometer card is used to support the child to indicate the interference score (0=totally not, 8=very much) representing his or her subjective level of daily life impairment. In a pilot study, the adapted ADIS-C PTSD section appeared both feasible and child friendly and had excellent interrater reliability with kappa (κ) varying between .87 and 0.95 (Mevissen et al., 2014).

#### CBCL – Dutch version

The Dutch version of the CBCL (Achenbach, 1991; Verhulst, Van der Ende, & Koot, 1996) was used to determine convergent validity. The CBCL is a widely used 113-item behavior rating scale for children aged 6–18 years to measure emotional and behavioral problems. Caregivers rate their child's behavioral and emotional problems on a 3-point Likert-type scale (0=absent, 1=occurs sometimes, 2=occurs often). For the Dutch version of the CBCL, good reliability and validity have been demonstrated, also for children with ID (Douma, Dekker, Verhulst, & Koot, 2006; Verhulst et al., 1996).

In the present study, Cronbach's alpha's of the CBCL internalizing as well as the externalizing scale was 0.91 (excellent) (Bakeman & Gottman, 1986).

### Procedure

Between April 2012 and June 2014, children with their primary caregivers who were referred to an outpatient center of a psychiatric service in the eastern part of the Netherlands received an information brochure and were asked to participate in the study. Eighty caregivers and their children (aged 12 or older) gave their written informed consent, permission to record the interview on video, and permission to process the data anonymously. This study was performed in accordance with the precepts and regulations for research as stated in the Declaration of Helsinki, and the Dutch Medical Research on Humans Act (WMO) concerning scientific research. The WMO was not applicable to the present study because (1) the surveys contained only a small number of items, (2) history taking by a psychologist, including potentially traumatic events, is common practice in an outpatient center in child and adolescent psychiatry, (3) the study lacked random allocation, and (4) no “physical infringement of the physical and/or psychological integrity of the individual” was to be expected.

Trained psychologists administered the interviews for the children and the primary caregiver(s).

While the children were being interviewed, the primary caregiver(s) filled out the CBCL. All interviews were recorded on video.

Three children did not complete the interview. Two of them did not understand the questions and one child became upset when asked the first question of the event section. This child was not able to concentrate on the questions that followed.

PTSD criteria were applied to child (*n*=77) as well as caregiver interviews (*n*=80) using DSM-IV-TR and DSM-5. The first author and two psychologists independently coded the symptom questions according to DSM-IV-TR and DSM-5 diagnostic criteria, and in case of disagreement, the last author made the final decision. The same procedure was followed with regard to the decision on whether or not an event met the Criterion A.

For DSM-5, two different analyses were performed. First, data of all participants were scored according to DSM-5 criteria and according to DSM-5 criteria for children aged 6 years and younger. Second, DSM-5 criteria were applied for child and caregiver data taking into account the child's estimated mental age (eMA) [(IQ/100) * chronological age—max 16 years]. Fifty-six children had an eMA of ≥7 years and 24 children had an eMA <7 years.

### Statistical analyses

*T*-tests for independent samples, Mann–Whitney tests, Chi-square and Fisher's exact tests, Cohen's Kappa, and Pearson's correlations were performed. All tests were two-tailed and the level of significance was set at 0.05.

## Results

### Interrater reliability

Three secondary observers independently scored 25% of the interviews (20 child and 20 caregiver interviews) on a question-by-question basis (63 questions) with results corrected for chance. Interrater agreement was 90%. Mean Cohen's kappa of both the child interviews and the caregiver interviews was excellent (Bakeman & Gottman, 1986) (child: κ=0.81, range: 0.38–1, *M*=0.81, SD=0.16; caregiver: κ=0.79, range: 0.34–1, *M*=0.79, SD=0.15).

### Correspondence of PTSD symptoms in children with MBID with those included in the DSM-IV-TR and DSM-5 PTSD algorithms (also see Table 1)

Eighty-nine times an open-ended symptom question was answered in the affirmative. Some answers to the subsequent question “What do you notice” were vague, for example, “She has changed.” Clear answers were compared with the PTSD symptoms already included in the interview. All of these appeared to match with symptoms already included in the interview. For example, the answer “Jitters in my stomach” matched with the symptom question “If something reminds you of the event(s), do you get awful feelings in your body?” Next, it was checked whether the participant had really answered “yes” to that corresponding interview question. If not, it was checked whether the child may have been unfairly diagnosed as not having a PTSD diagnosis (i.e., false negative). In one out of the 157 scored interviews, this might have been the case.

**Table 1:** ADIS-C PTSD MBID symptom section^a^

	Symptom questions (answer categories: yes, no, other^b^)	DSM-IV-TR	DSM-5	DSM-5Y^c^	Atypical	Kappa
28	Do you still often think of the event(s) even though you really don't want to? If eMA^d^<7: Do you sometimes play or draw what happened?	x	x	x		0.90
29	Do you hear voices in your head about the event (s)?	x				0.88
30	Do you frequently have nightmares or horrible dreams about what has happened?	x	x	x		0.71
31	Do you have nightmares or horrible dreams about other things?	x	x	x		0.82
32	Do you sometimes feel as if it could happen again right now?	x	x	x		0.90
33	Do you get totally upset if something reminds you of those event(s)?		x	x		0.68
34	Do you start to act in a very happy way if you have to think about the event(s)?				x	0.73
35	If something reminds you of the event(s), do you get awful feelings in your body? For example, does your heart start to beat much faster, do you start to sweat or shake?	x	x	x		0.80
36	If something reminds you of the event(s) do you get stomachache or headache?	x	x	x		0.90
37	If something reminds you of the event(s) do you notice anything different about yourself?					1.00
38	Do you try as hard as you can, not to think of those event(s)?	x	x			0.90
39	Do you try to stay away from things that remind you of the event(s)? For example, situations, places, noises, smells?	x	x	x		1.00
40	Are there some parts of the event(s) you no longer remember?	x	x			0.79
41	Since those event(s) happened, did you stop doing things you really liked to do before, for example, playing games or going out, hobbies? Or do you no longer like to do those things?	x	x	x		0.63
42	Do you no longer feel like seeing your friends or girlfriends since the event(s)?	x	x	x		0.78
43	Do you feel lonely or isolated more often since those event(s)?	x	x	x		0.85
44	Since the event(s), has it become more difficult for you to show other people how you feel? For example, do you avoid showing someone else how you are feeling and do you keep your feelings to yourself?	x	x			0.92
45	Has it become more difficult to trust other people since the event(s)?		x			1.00
46	Do you think that if you are grown up, you would be able to do anything you would like to do, for example, receive training, get married, find a job, raise children, or any of these types of things?	x	x			0.90
47	Do you often feel bad? Do you, for example, often have feelings of anxiety, blame, or shame, or do you often think things are very awful?		x	x		0.89
48	Do you always blame yourself or others about what has happened while in fact this is not with good reason?		x	x		0.88
49	Can't you feel happy anymore since those event(s)?	x	x	x		0.73
50	Is it as if you can't feel anything anymore since those event(s)?		x			0.38
51	Did you start doing things again you didn't do since you were a little child, for example, wetting your pants again, sucking your thumb, or always trying to stay close to your father and mother or caregivers?					0.74
52	Are you unable to sleep well, for example, is it difficult to fall asleep, do you often wake up during the night, or do you wake up too early in the morning?	x	x			0.62
53	Do you get angry more often since those event(s) happened?	x	x	x		0.67
54	Do you sometimes hurt yourself or others or do you break things?		x	x		0.83
55	Do you have serious outbursts of anger?	x	x	x		0.90
56	Is it difficult to keep your mind on things, do you have difficulties concentrating?	x	x	x		1.00
57	Do you always watch out very carefully because you think something bad might happen again?	x	x	x		1.00
58	Are you seriously frightened when something happens unexpectedly or suddenly, for example, if all of a sudden you hear a loud noise or if someone touches you unexpectedly?	x	x	x		0.92
59	Do you no longer watch out for what you're doing; do you act dangerously?		x			0.73
60	Since the event(s), did you change eating behavior, for example, eating too much or too little?				x	0.69
61	Do you no longer take care of yourself as well as you did before, for example, has it become more difficult to wash yourself and dress and do you no longer succeed in brushing your teeth well?				x	1.00
62	Since those events, is it harder to accept when things go different than expected, for example, if an appointment has been cancelled or if you suddenly have to do something unexpected?				x	0.73
63	Do you have to do some things again and again or always in the same order?				x	0.60
64	Have you noticed anything else that's different about yourself since the event(s)?					0.85

a Caregivers were asked the question for the child, for example, “Does (child's name) still often think of the event(s) even though he/she really does not want to?”

b The child answers, for example, “I don't know,” “sometimes,” or any other unclear answer.

c DSM-5 6 years and younger.

d eMA=estimated mental age [(IQ/100) * chronological age—max 16 years].

Furthermore, it was examined whether the five interview questions that are not included in DSM-IV-TR, DSM-5, and DSM-5 for children 6 years and younger (eating problems, decreased self-care, difficulties when things go differently than expected, obsessive-compulsive behaviors, and pretending to be happy) might be distinctive for children with MBID, the so-called atypical PTSD symptoms. It was found that, except for caregiver reports of children with an eMA <7 years, the mean number of those five symptoms was significantly higher in children who met the full PTSD symptom criteria than in children who did not meet the full PTSD symptom criteria, irrespective of the PTSD algorithm and whether child or caregiver data were used (subsequent statistical outcomes are available upon request).

### Association between level of exposure to potentially traumatic events and fulfilling PTSD symptom criteria

Table 2 presents the differences in mean number of potentially traumatic events between children who did and those who did not meet full PTSD symptom criteria according to the different PTSD algorithms for child as well as caregiver reports.

**Table 2:** Mean number of potentially traumatic events between children who fulfilled and those who did not fulfill PTSD symptom criteria, according to the different diagnostic algorithms and child and caregiver reports

Diagnostic algorithm	PTSD symptom criteria	Reported by the child						Reported by the caregiver					
	
		*n*	*M*	*SD*	*t*	*df*	*p*	*n*	*M*	*SD*	*t*	*df*	*p*
DSM-IV-TR	Yes	20	14.55	3.82	5.40	75	0.000***	21	11.33	3.60	1.26	78	0.211
	No	57	9.09	3.92				59	10.24	3.36			
DSM-5	Yes	18	13.56	5.20	3.47	75	0.001**	26	12.42	3.09	3.69	78	0.000***
	No	59	9.58	3.94				54	9.61	3.24			
DSM-5 for children 6 years and younger	Yes	26	12.92	4.68	3.57	75	0.001**	35	12.29	2.87	4.51	78	0.000***
	No	51	9.27	4.01				45	9.16	3.23			
DSM-5 children eMA^a^ ≥7 years	Yes	11	15.18	4.64	3.58	54	0.001**	16	12.94	2.67	3.59	54	0.001**
	No	45	10.16	4.06				40	9.93	2.90			
DSM-5 children eMA^a^ <7 years	Yes	10	10.50	4.58	1.93	19	0.069	13	12.15	2.88	3.61	22	0.002**
	No	11	7.27	3.00				11	7.27	3.74			

*Note*. *p<0.05,

** *p*<0.01,

*** *p*<0.001.

a eMA=estimated mental age: (IQ/100)×age (age max=16×12 months).

Children who met the full PTSD symptom criteria had been exposed to a significantly greater number of potentially traumatic events than those not meeting PTSD criteria, except when DSM-IV-TR was applied to caregiver reports and DSM-5 for children 6 years and younger was applied to child reports of children with an eMA <7 years.

### Applicability of the criterion A for trauma

Table 3 presents results of Chi-square tests on the association between meeting Criterion A and fulfilling PTSD symptom criteria for each of the PTSD algorithms.

**Table 3:** PTSD Criterion A and PTSD symptom criteria for DSM-IV-TR, DSM-5, and DSM-5 for children 6 years and younger, according to child and caregiver reports

PTSD Criterion A		PTSD symptom criteria Reported by the child			PTSD symptom criteria Reported by the caregiver		
		
				*p*			*p*
		Yes	No		Yes	No	
DSM-IV-TR	Yes	20	42	0.018*	19	49	0.502
	No	0	15		2	10	
DSM-5	Yes	15	35	0.090	23	33	0.023*
	No	3	24		3	21	
DSM-5 for children 6 years and younger	Yes	20	26	0.048*	30	24	0.003**
	No	6	25		5	21	
DSM-5 children eMA^a^ ≥7 years	Yes	10	30	0.150	15	27	0.084^b^
	No	1	15		1	13	
DSM-5 children eMA^a^ <7 years	Yes	6	3	0.198	10	4	0.095
	No	4	8		3	7	

*Note*.

* *p*<0.05,

** *p*<0.01.

a eMA=estimated mental age: (IQ/100)×age (age max=16×12 months).

b Fisher's exact test indicated significance (*p*=0.047) and thus did not corroborate the finding of the chi-square test.

Children who had been exposed to a Criterion A event were more likely to meet PTSD symptom criteria than those not exposed to a Criterion A event. This outcome held true for child reports of DSM-IV-TR and DSM-5 for children 6 years and younger, and for the caregiver reports of DSM-5 and DSM-5 for children 6 years and younger.

Children who met full PTSD symptom criteria, but who did not meet Criterion A for the specific PTSD algorithm, had a history of event(s) fitting DSM-IV-TR A criterion and/or event(s) that were potentially A events but reports contained insufficient information to score the event(s). The only child who met PTSD DSM-5 and DSM-5 6 years and younger symptom criteria without reporting a type A event had an eMA of 5.3 years and was referred to the outpatient center with suspicion of autism. According to the caregiver report, a PTSD diagnosis was not applicable. Twice a caregiver reported no type A event while symptoms met DSM-5 PTSD criteria. In one case, the father, with whom the relationship was close, left the family when the child was 3 years old. Moreover, the child had a history of being bullied. In the second case, parents divorced when the child was 2 years old, with subsequent foster placement of the child. The caregiver reported “suspected” abuse, so the answer could not be coded as a type A event.

### Association between subjective level of impairment and fulfilling PTSD symptom criteria

Table 4 presents the differences in mean thermometer scores of children fulfilling the PTSD criteria according to the different PTSD algorithms and children not fulfilling these symptom criteria, according to child and caregiver reports.

**Table 4:** Mean thermometer scores in children who fulfilled and those who did not fulfill PTSD symptom criteria, according to the different diagnostic algorithms and child and caregiver reports

Diagnostic algorithm	PTSD symptom criteria	Reported by the child						Reported by the caregiver					
	
		*n*^a^	*M*	*SD*	*t*	*df*	*p*	*n*^a^	*M*	*SD*	*t*	*df*	*p*
DSM-IV-TR	Yes	20	6.35	1.70	6.12	55.88	0.000***	17	6.71	1.11	5.29	59.02	0.000***
	No	56	3.09	2.80				63	4.56	2.43			
DSM-5	Yes	16	6.94	1.57	7.23	59.02	0.000***	23	6.43	1.16	4.86	76.41	0.000***
	No	60	3.15	2.69				57	4.44	2.51			
DSM-5 for children 6 years and younger	Yes	19	6.58	1.68	6.64	51.06	0.000***	30	6.37	1.19	5.15	74.45	0.000***
	No	57	3.07	2.73				50	4.20	2.55			
DSM-5 children eMA^b^ ≥7 years	Yes	11	6.91	1.58	5.72	54	0.000***	15	6.33	1.23	3.75	49.66	0.000***
	No	45	2.58	2.38				41	4.41	2.56			
DSM-5 children eMA^b^ <7 years	Yes	5	7.00	1.73	1.98	11.98	0.071	10	6.60	0.97	3.26	17.92	0.004**
	No	15	4.87	2.90				14	4.21	2.49			

*Note*. As the assumption of equal variances was not met (tested with Levene's test) results of *t*-tests for unequal variances are reported. *p<0.05,

** *p*<01,

*** *p*<001.

a One thermometer score was missing.

b eMA=estimated mental age: (IQ/100)×age (age max=16×12 months).

Except for reports from children with an eMA<7 years, children who met the PTSD symptom criteria of DSM-IV-TR, DSM-5, and DSM-5 for children 6 years and younger reported a significantly higher mean thermometer score than those who did not meet the PTSD symptom criteria.

### Association between PTSD symptom scores and CBCL scores

A significant positive correlation was found between total number of PTSD symptoms and CBCL internalizing subscale score (DSM-IV-TR: *r*=0.53, *p*<0.01; DSM-5: *r*=0.58, *p*<0.01; DSM-5 6 years and younger: *r*=0.57, *p*<0.01), as well as CBCL externalizing subscale score (DSM-IV-TR: *r*=0.23, *p*<0.05); DSM-5: *r*=0.29, *p*<0.01; DSM-5 6 years and younger: *r*=0.26, *p*<0.05). Also for children with an eMA<7 years, a significant positive correlation (DSM-5 6 years and younger: *r*=0.65, *p*<0.01) was found between the total number of PTSD symptoms and the CBCL internalizing subscale score. For the CBCL externalizing subscale score, the correlation with the total number of PTSD symptoms was positive though not significant (*r*=0.25, *p*=0.24) for children with an eMA<7 years.

## Discussion

The present study is the first study to validate a PTSD clinical interview for assessing PTSD in children with MBID. Both the child and caregiver version of the interview yielded excellent interrater reliability, and proved to have good convergent validity for assessing PTSD.

It was found that PTSD does not manifest itself atypically in children with MBID. PTSD symptoms reported by children with MBID and their caregivers were in accordance with the PTSD symptoms in the DSM-IV-TR and DSM-5. This finding not only is in line with the results of the pilot study by Mevissen et al. (2014), it also underpins the expert guidelines for the assessment of PTSD in people with mild ID as recommended in the Diagnostic Manual-Intellectual Disability (DM-ID) (Fletcher, Loschen, Stavrakaki, & First, 2007). The five atypical symptoms that were included in the interview were more likely to be recognized by children and caregivers whose reports were meeting PTSD criteria than by participants whose reports did not fulfill all PTSD criteria. Most of these atypical symptoms may be similar to symptoms of depression and anxiety, seen in children with severe or “complex” forms of PTSD (e.g., Suliman et al., 2009).

According to DSM-IV-TR analyses of the child reports, children who had been exposed to a Criterion A event would be more likely to meet PTSD symptom criteria than children who had not been exposed to a Criterion A event. However, the caregiver data did not correspond with those of the children. This is conceivable given that caregivers are only partially able to assess the inner world and perceptions of their trauma-exposed child (Criterion A2). The DSM-IV-TR differed from the DSM-5 in that children reported symptoms fulfilling the DSM-5 PTSD symptom criteria in the absence of a DSM-5 type A event. This finding might be explained by the sharpened formulation of the criterion of what constitutes a type A event as introduced in DSM-5 in comparison to former DSM A1 definitions. That many of the children who met the Criterion A did not meet PTSD symptom criteria corresponds well with other child trauma samples (Alisic, Jongmans, Van Wesel, & Kleber, 2011).

Children meeting PTSD symptom criteria were found to report a higher level of exposure to potentially traumatic events than those not meeting PTSD symptom criteria. This held true for all PTSD algorithms, and for caregiver as well as child reports. This finding is in line with the general literature about PTSD, and with studies showing that the likelihood of developing PTSD is linearly associated with the level of exposure to traumatic events (Perkonigg, Kessler, Storz, & Wittchen, 2000; Wilker et al., 2015).

Meeting PTSD symptom criteria appeared to be associated with higher subjective levels of daily life impairment, regardless of the algorithm that was used and held true for child as well as caregiver reports. Apparently, elevated distress and impairments in daily life functioning are characteristic of PTSD, irrespective whether a child has ID or not.

Positive correlations were found between rates of PTSD symptoms and CBCL scores. The correlations were higher for internalizing problems than for externalizing problems. This seems logical because PTSD largely consists of symptoms representing thoughts and feelings included in the PTSD clusters re-experiencing, avoidance, and negative alterations in mood and cognition. In clinical practice, the CBCL is used to assess psychopathology in children with MBID. An internalizing CBCL score in the deviant range should be a sign for psychologists to further investigate potential psychological trauma.

## Strengths and limitations

The study has several strengths and limitations. Obvious strengths were that it was the first study to validate a PTSD clinical interview for children with MBID in which three PTSD algorithms were compared, taking ID into account. Considering the latter, recently Gigengack, Van Meijel, Alisic, and Lindauer (2015) demonstrated the importance of the developmentally sensitive PTSD criteria for young children, as incorporated in the DSM-5 subtype for children aged six years and younger. In the present study, these DSM-5 6 years and younger criteria were used for children having a mental age corresponding with that of non-disabled children aged six years and younger. This PTSD subtype does not include symptoms that require skills which young children have not yet developed, such as verbal expression, memory, or abstract thought, thereby improving the identification of PTSD relative to DSM-IV-TR for this (mental) age category. Taking into account the limited skills of children with a mental age of six years and younger, it is also a strength of this study that both child and caregiver data were collected. Furthermore, the thorough event section of the interview seems to be valuable considering that there is evidence indicating that the number of traumatic event types experienced leads to the best prediction of lifetime PTSD (Wilker et al., 2015). A feature of the study which is difficult to qualify in terms of strength or limitation is the use of a timeline which incorporates all negative events the participant has been exposed to considering it is common practice to take into account only one event when asking for trauma-related symptoms. Research findings suggest that traumatic events, experienced during developmental sensitive periods, have a significant impact upon the development of childhood and adult psychopathology (Wilker et al., 2015). From this point of view, assessment of trauma exposure with use of a timeline seems to be valuable. Limitations of this study were that IQ data were based on information from case files and that children with IQ 70–85 (i.e., borderline ID) were overrepresented. Additional analyses revealed that the pattern of results found for children with IQ 70–85 was comparable for the sub-sample of children with IQ 50–70 with regard to symptom severity, level of exposure to potentially traumatic events, as well as subjective level of daily life impairment (Supplementary Tables). It could be argued that the sample is not fully representative of the overall population of children with MBID because of self-selection, meaning that replication is needed with additional samples of children with MBID. It is worth noting that the participants of the current study were referred to the outpatient psychiatric service under the suspicion of a wide variety of psychiatric disorders by a wide variety of notifying parties.

## Concluding comments and future directions

The adapted ADIS-C as a valid and reliable PTSD clinical interview could be of great relevance in mental health care for children and adolescents with MBID. Timely detection and diagnosis in this population, which is at higher risk for exposure to potentially traumatic events and developing PTSD, is the first step in preventing serious long-term psychological and somatic disorders that have been found to require costly professional care (Olff, 2015). This could be enhanced by the development of a less time-consuming screening tool to identify children with MBID who need further clinical assessment by trained professionals. The present study focused on children. An important future direction of research is the development and validation of a PTSD clinical interview for adults with MBID, an even larger and likewise at high-risk target population.
